# Simultaneous Wastewater Treatment and Resources Recovery by Forward Osmosis Coupled with Microbial Fuel Cell: A Review

**DOI:** 10.3390/membranes14020029

**Published:** 2024-01-23

**Authors:** Hengliang Zhang, Liang Duan, Shilong Li, Qiusheng Gao, Mingyue Li, Fei Xing, Yang Zhao

**Affiliations:** 1 State Key Laboratory of Environmental Criteria and Risk Assessment, Chinese Research Academy of Environmental Sciences, Beijing 100012, China; 201831470030@mail.bnu.edu.cn (H.Z.); slone_li@126.com (S.L.); 201931470030@mail.bnu.edu.cn (Q.G.); lmy080510@163.com (M.L.); feixgy@126.com (F.X.); zhaoyang@craes.org.cn (Y.Z.); 2 Basin Research Center for Water Pollution Control, Chinese Research Academy of Environmental Sciences, Beijing 100012, China; 3 College of Water Sciences, Beijing Normal University, Beijing 100875, China

**Keywords:** osmotic microbial fuel cells, cooperation mechanisms, resources recovery, operational factors, potential applications and challenges

## Abstract

Osmotic microbial fuel cells (OsMFCs) with the abilities to simultaneously treat wastewater, produce clean water, and electricity provided a novel approach for the application of microbial fuel cell (MFC) and forward osmosis (FO). This synergistic merging of functions significantly improved the performances of OsMFCs. Nonetheless, despite their promising potential, OsMFCs currently receive inadequate attention in wastewater treatment, water reclamation, and energy recovery. In this review, we delved into the cooperation mechanisms between the MFC and the FO. MFC facilitates the FO process by promoting water flux, reducing reverse solute flux (RSF), and degrading contaminants in the feed solution (FS). Moreover, the water flux based on the FO principle contributed to MFC’s electricity generation capability. Furthermore, we summarized the potential roles of OsMFCs in resource recovery, including nutrient, energy, and water recovery, and identified the key factors, such as configurations, FO membranes, and draw solutions (DS). We prospected the practical applications of OsMFCs in the future, including their capabilities to remove emerging pollutants. Finally, we also highlighted the existing challenges in membrane fouling, system expansion, and RSF. We hope this review serves as a useful guide for the practical implementation of OsMFCs.

## 1. Introduction

There has been a surge in the global demand for water and energy in recent years due to rapid economic development, growing populations, and escalating environmental pollution [1]. The increased demands lead to a shortage of fresh water and energy resources, thus posing challenges to human survival. It was estimated that severe water scarcity would affect approximately 2.7 billion people in 20% of countries and regions by 2025 [2]. The imbalance between water supply and demand is already significant, with the latter expected to overshoot the former by 40% by 2030 [3]. Simultaneously, fossil fuels such as coal, oil, and natural gas will exhaust gradually in 114, 50, and 52 years, respectively [2]. To address the increasing demands, access to sustainable and renewable water and energy sources are crucial. Wastewater is a rich source of various valuable resources, with each gram of chemical oxygen demand (COD) containing approximately 17.8–28.7 kJ of energy [4]. Among these resources, nutrients, energy, and water (NEW) are critical and can be recovered through various wastewater treatment technologies [5], including microbial fuel cells (MFCs) [6], hydrothermal gasification (HTG) [7], and aqueous phase reforming (APR) [8] for the recovery of energy from high-energy wastewater, membrane technology for water recovery, and struvite for recovering nutrients [9]. However, these separate technologies have difficulty in recovering NEW from wastewater simultaneously. Therefore, there is an urgent requirement for a novel treatment technology that can achieve both resources recycling and energy recovery from wastewater, thereby addressing the water–energy nexus effectively.

FO technology is a membrane technology that spontaneously transports water from the feed solution across a membrane to the draw solution. It is driven by the osmotic pressure difference on both sides of the FO membrane, thereby achieving the interception of pollutants and the extraction of high-quality water [10,11]. Due to the lack of an external energy input, FO technology is an energy-saving technology with great appeal [12,13]. In recent years, MFC has emerged as a wastewater treatment technology, which can simultaneously achieve pollutant degradation and energy recovery. This technology falls under the category of bio-electrochemical systems, which also includes microbial electrolysis cells (MECs) and microbial desalination cells (MDCs) [14]. The electricity generation in MFCs is based on the principle that electroactive microorganisms can transform the energy stored in the chemical bonds of organic matter into electricity via electron transfer processes [15]. The configurations of MFCs can be categorized into single-chamber, dual-chamber, and multi-chamber models [16,17]. The typical MFCs are dual-chamber, consisting of an anode chamber and a cathode chamber separated by a proton exchange membrane (PEM). Within the anode chamber, microorganisms oxidize organic matter in an anaerobic environment to produce electrons and protons, then the electrons transmit from the anode electrode to the cathode electrode via an external circuit, while the protons move from the anode chamber to the cathode chamber through the PEM. In the cathode chamber, the transported electrons and protons react with electron acceptors such as oxygen to produce water [18,19]. MFC has been widely used to treat varieties of wastewater including domestic, livestock, pharmaceutical, food wastewater, as well as urine [20,21,22,23]. Although MFC has been a novel alternative technology for wastewater treatment, there are significant limitations, such as high investment costs and the requirement for further treatment of effluent prior to discharge [24].

OsMFCs combine the advantages of the traditional MFCs and FO technology. Essentially, the PEM in MFCs is replaced by an FO membrane, thereby endowing MFCs with the function of producing high-quality water. Consequently, OsMFCs can simultaneously achieve wastewater treatment, electricity generation, and high-quality water production [25,26]. The configurations of the OsMFCs mirror that of traditional dual-chamber MFCs, comprising an anode chamber, anode solution (FS), FO membrane, cathode chamber, cathode solution (DS), and electrodes. The anode and cathode electrodes are typically carbon brushes, carbon cloth, etc., and they connect via wires to form an external circuit. OsMFCs share power generation principles with the traditional MFCs [27,28]. In the OsMFCs, anolyte (FS) is typically low-conductivity organic wastewater with lower osmotic pressure, while the catholyte (DS) is high-concentration saline water with higher osmotic pressure. The osmotic pressure difference between the anolyte and catholyte facilitates the spontaneous migration of water from the anode chamber to the cathode chamber through the FO membrane. This process results in concentration of the anolyte (FS) and the dilution of the catholyte (DS) [5,29]. Subsequently, the diluted draw solution can produce high-quality water and undergo regeneration of the DS using reverse osmosis (RO) technology.

**Figure 1:**
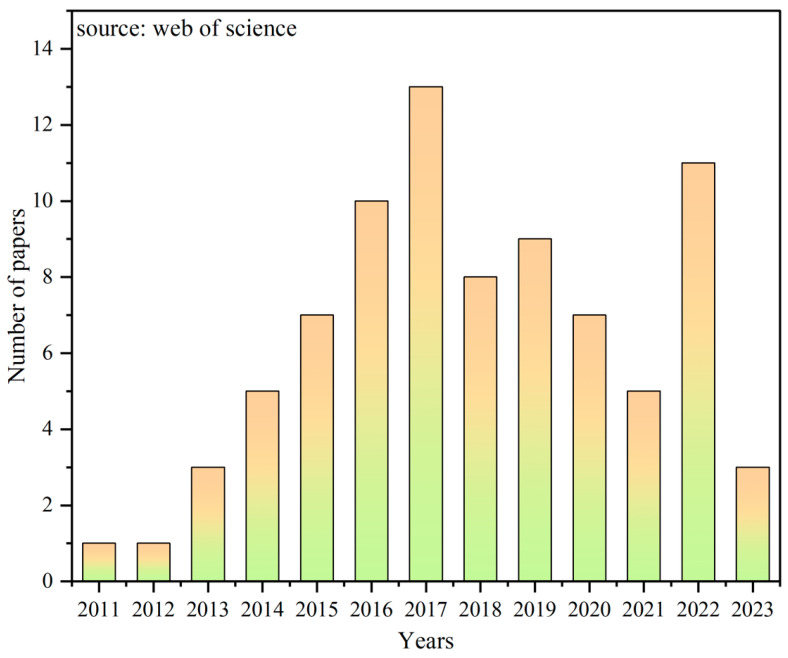
Number of published papers about OsMFCs. Source: Web of Science in December 2023. Topics: “forward osmosis” and “microbial fuel cells”.

The concept of OsMFCs was first proposed by Zhang in 2011 [30] and then attracted widespread attention quickly due to its multifaceted capabilities of degrading pollutants, producing high-quality water, and generating electricity. A total of 83 related papers were retrieved through the Web of Science database in December 2023 with the search topics of “forward osmosis” and “microbial fuel cells”. These papers were cited a total of 2771 times, with an average citation frequency of 33.39 per paper and an h-index of 33. Figure 1 illustrated the annual volume of published papers, showing a notable upward trend prior to 2017, and peaking with 13 publications in that year. Following a minor dip, papers published on the topic surged to 11 in 2022. This trend indicated that OsMFCs remain a research hotspot over the past 2 years. Among all publications, *Journal of Membrane Science*, *Bioresource Technology*, *Chemical Engineering Journal*, and *Membrane* ranked joint first with 7 publications each, followed by *Water Research, Journal of Cleaner Production*, and *Desalination*. Additionally, the United States and China dominated the number of articles.

Owing to their abilities to concurrently remove pollutants, generate electricity, and produce high-quality water, OsMFCs have been used in various domains such as domestic sewages [28], garbage leachates [31], and petroleum wastewater treatment [32]. As a result, they have emerged as a potential solution to modern energy needs and environmental challenges. Despite the existence of several overview articles about OsMFCs [2,5,24,29], these articles were only slightly involved and were not comprehensive, lacking specialized, systematic, and comprehensive reviews on OsMFCs. This article offers a systematic and comprehensive review of OsMFCs to fill this void, tracing developments from their inception in 2011 to the present day. Firstly, the cooperation mechanisms of MFC and FO are described in detail in OsMFCs. Secondly, the applications in resources recovery are introduced systematically. Then a comprehensive review of the operational factors is provided. Finally, the challenges encountered in practical applications and potential applications are prospected. Through this comprehensive review of the OsMFCs, the paper aims to foster future applications by providing guidelines.

## 2. Cooperation Mechanisms

**Figure 2:**
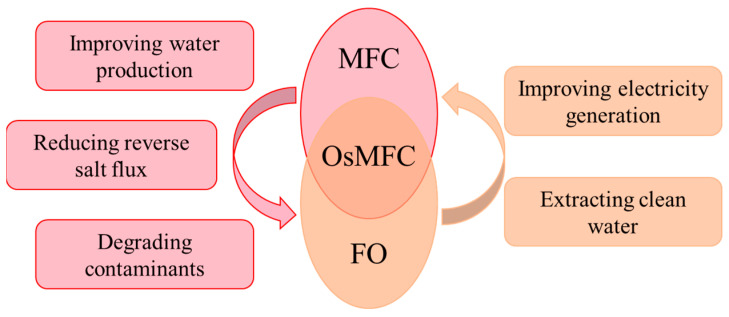
Cooperation mechanisms between MFC and FO in the OsMFC system.

The incorporating FO membrane as the separator in the MFC exhibited mutual benefits for both the FO process and the MFC system, and their cooperation mechanisms are shown in Figure 2. Regarding FO processes, the integration MFC could improve water production, reduce RSF, and degrade contaminants in wastewater. For the MFC, the incorporation of the FO membrane could enhance electricity generation performance while simultaneously facilitating the direct extraction of clean water. This novel cooperation between FO and MFC technologies holds great promise for future applications in sustainable energy and environmental remediation.

### 2.1. MFC Enhanced FO Performance

#### 2.1.1. Improving Water Flux

**Figure 3:**
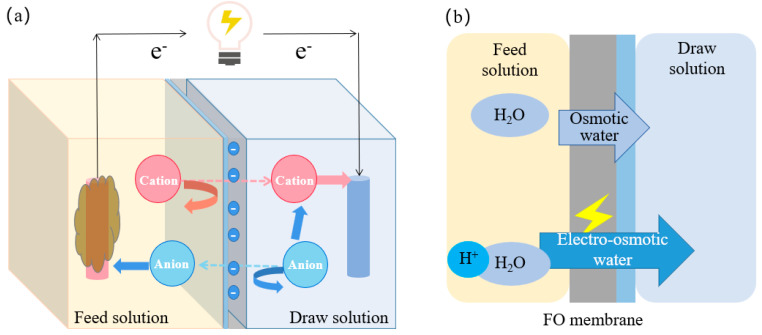
Principle of increasing water flux (**a**) mechanism of RSF inhibition (**b**) in OsMFCs.

Compared with MFC, the incorporation of FO membrane could enable the extraction of clean water. This was achieved through the osmotic pressure difference between the high-concentration anolyte and the low-concentration catholyte, facilitating the forward transport of water [33]. Additionally, the presence of an electric current promoted the osmosis process of water molecules. Protons formed hydrated protons through hydrogen bonding with free water molecules [34]. In the OsMFCs system, electrons transferred from the anode to the cathode through an external circuit, which increased the electronegativity of the cathode [35]. The generation of electric current intensifies the electrostatic forces and potential difference, thereby increasing the flux of hydrated protons (free water) due to the effect of electro-osmosis (Figure 3a). As a result, MFC played a further role in enhancing the water production capability of FO systems.

However, it was important to note that a higher current density does not necessarily lead to an increase in electro-osmotic water flux. Zhao et al. [36] observed that as the current density increases, the water flux decreased. They found that at current densities below 4.86 A·m^−2^, the electro-osmotic water passing through the FO membrane exceeded the osmotic water. The phenomenon might be attributed to polarization effects that occurred at high current densities. To further understand the mechanisms of electro-osmosis and resolve the negative influences of high current density on water flux in OsMFCs, more research was needed. This would help optimize OsMFCs for efficient water extraction and electricity generation.

#### 2.1.2. Reducing Reverse Salt Flux

Reverse salt flux (RSF) was a challenge in FO processes. It could result in negative consequences such as diminished effluent quality, loss of draw solute, and increased operational costs [37,38]. However, several studies have demonstrated a significant inhibition of RSF when coupling MFCs with FO technology [39]. The presence of current in the OsMFCs played a crucial role in inhibiting the reverse migration of cations. When there was current in the system, the reverse migration of cations could be effectively inhibited. In OsMFCs, electrons flowed from the anode to the cathode through an external circuit, cations diffusion from the draw solution to the feed solution were reduced to maintain electrical neutrality. In fact, cations on the anode side could be attracted toward the cathode [35,40]. On the other hand, as the flowing of electrons, the anode required anions to maintain charge balance. Theoretically, this could lead to increased RSF of anions in the draw solution. However, the negative charge of the FO membrane impeded the reverse diffusion of anions. Consequently, coupling MFCs led to the inhibition of RSF for both anions and cations in FO [41] (Figure 3b). In addition, controlling the current density could adjust the RSF. Higher current densities have been shown to decrease the RSF of negatively charged Thin-Film Composite (TFC) membranes in OsMFCs [39]. This could be attributed to the electrostatic repulsion force, which played a crucial role in the ion permeability of TFC membranes. The increased current density inhibited the electrostatic repulsion force, thereby reducing RSF.

#### 2.1.3. Degrading Contaminants

FO have demonstrated the potential for extracting clean water from wastewater [42,43]. However, FO processes primarily involved physical processes without chemical or biological reactions. Due to the increasing complexity and diversity of pollutants in wastewater, FO alone might not degrade the pollutants efficiently. When coupled with Microbial Fuel Cells (MFCs), OsMFCs could leverage the catalytic capabilities of microorganisms to facilitate oxidation-reduction reactions and effectively degrade pollutants. OsMFCs have demonstrated their ability to remove various pollutants from wastewater. For example, they could achieve a removal efficiency of over 70% for ammonia nitrogen and total nitrogen [31], a COD removal rate of 63 ± 8% when treating raw domestic wastewater [44], and up to 90% removal when using acetic acid as a carbon source [45]. In the study of Cao et al. [46], the removal effect of OsMFCs on hexavalent chromium [Cr (VI)] was investigated for the first time, and the results showed that the removal efficiency of Cr (VI) was 97.6% when 0.2 M NaCl/Cr (VI) was employed as draw solute at pH 2. In addition to conventional pollutants, OsMFCs also exhibited promising potential for the removal of complex organic pollutants. While studies on the degradation of such pollutants using OsMFCs were limited, sufficient research existed on MFCs, due to the similar principles. Cheng et al. investigated the removal efficiency of MFCs for sulfonamides, achieving a removal rate of over 99% for sulfamethoxazole, 66.91% for sulfadiazine, and 67.21% for sulfamethazine [47]. Cylindrical MFCs exhibited excellent removal rates for complex antibiotic wastewater at the milligram level [48], achieving removal rates close to 100% for chloromycin, sulfadiazine, roxithromycin, and norfloxacin. Despite the scarcity of current research on organic pollutant degradation by OsMFCs, such as antibiotics, further investigation and verification are required to fully understand their capabilities and optimize their performance in this regard.

### 2.2. FO Improved MFC Performance

#### 2.2.1. Improving Electricity Generation

**Figure 4:**
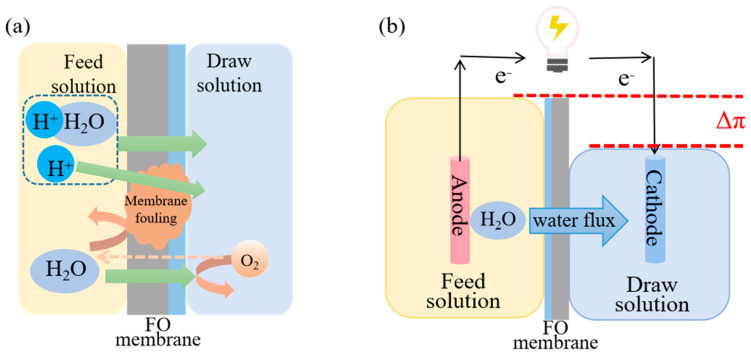
Electricity generation capacity improvement mechanism diagram (**a**) and principle of water extraction based on osmotic pressure difference in OsMFC (**b**).

In comparison to traditional ion exchange membranes (IEM), FO membranes exhibited a notable capability to facilitate the passage of water molecules. Importantly, the water flux through FO membranes concurrently transported ions (such as protons) from the anode to the cathode, thus accelerating the transportation of ions across the membrane [49,50] (Figure 4a). Internal resistance was reduced with the FO membrane compared to the IEM, resulting in a higher maximum power generation (43 W/m^3^) than that of AEM (40 W/m^3^) and CEM (23 W/m^3^). Furthermore, the FO membrane and water flux could inhibit oxygen diffusion to the anode, minimizing adverse effects on anode electrogenic bacteria [49]. These combined effects significantly enhanced the electrical generation performance of OsMFCs compared to conventional MFCs [50,51].

Interestingly, membrane fouling in OsMFCs produced unexpected effects when compared to conventional MFCs. In conventional MFC reactors, membrane fouling of the IEM typically decreased electrochemical performance [52,53]. However, studies on OsMFCs revealed that membrane fouling led to a reduction in water flux without affecting electricity generation [45]. Zhu et al. discovered that membrane fouling increased the flux of protons and other ions, consequently reducing the internal resistance of the reactor [54]. Additionally, Zhao et al. observed that the hydraulic resistance coefficient of the fouling membrane increased by 4.97 times compared with the initial value by constructing a fouling membrane transfer model, while the salt mass transfer resistance coefficient remained largely unchanged [55].

#### 2.2.2. Extracting Clean Water

In order to balance the osmotic pressure difference across the membrane, FO exhibited selectivity in allowing water molecules to permeate into the higher concentration of the DS, thus enabling the extraction of clean water [33] (Figure 4b). Water extraction was a prominent feature of OsMFCs. For example, an OsMFC achieved water flux of 3.94 ± 0.22 L m^−2^ h^−1^ (LMH) with a catholyte containing 116 g NaCl/L, while there was no obvious water flux in a conventional MFC [30]. During a 10 h test, approximately 15 mL of water was added to the cathode, while no significant increase was observed in the MFC cathode. It was important to emphasize that water flux served as a fundamental and indispensable indicator for evaluating the performance of OsMFCs in all related studies. However, it was worth noting that the level of purity and quality of the extracted water could be influenced by various factors, including the characteristics of the influent wastewater, the design and operational parameters, and the conditions of the FO membranes.

## 3. Resources Recovery

### 3.1. Nutrient Recovery

The removal and recovery of nutrients has been successfully demonstrated in both MFCs and FO systems in previous research. For example, cations such as NH_4_^+^ in the anolyte (feed solution) were able to migrate to the catholyte through PEM due to the presence of current in MFCs system, and then an oxidation-reduction reaction occurred, resulting in an increase in pH and the release of ammonia gas [56]. The released ammonia could be recovered by an external sulfuric acid absorption bottle. Furthermore, phosphorus recovery was achieved by precipitation, capitalizing on the high pH of the catholyte (draw solution) [57]. In FO systems, nutrients would be concentrated along with the concentration of anolyte (feed solution) due to the presence of the FO membrane, enhancing the probability of recovery [58,59]. As an inheritor of MFC and FO, OsMFCs were expected to have the capability of nutrients recovery. Surprisingly, research on nutrients recovery is extremely limited at present by OsMFCs systems. Recently, some researchers have attempted to explore the potential of OsMFC for nutrients recovery. Qin et al. discovered that water flux and current were two important factors driving the transmembrane migration of ammonia nitrogen in the OsMFCs, consequently improving the removal rate of ammonia nitrogen [60]. With a water flux of 1.3 ± 0.2 LMH, the removal efficiency of ammonia nitrogen increased by 55.2 ± 6.5% compared to no water flux. Moreover, the removal efficiency of ammonia nitrogen increased from 40.7 ± 2.4% to 85.3 ± 3.5% when the current density increased from 0 to 1.8 ± 0.1 Am^−2^. Additionally, Gangadharan P et al. achieved a 99% retention rate of ammonia nitrogen in urine using NaCl as the DS and fresh urine as the FS in OsMFCs [61]. Although these studies did not recover ammonia, they greatly promoted the research progress of OsMFCs on nutrient recovery.

### 3.2. Energy Recovery

The electrochemical microorganisms in the anode chamber played a crucial role in converting chemical energy from organic pollutants into electricity in OsMFCs, thus enabling energy recovery from wastewater [28]. Table 1 summarizes the studies on energy recovery, revealing that OsMFCs could recover a portion of the energy as electricity. However, it is worth noting that there was also energy consumption during operation, primarily associated with the aeration of the cathode chamber. In the bio-electrochemical system (BES), researchers discovered that energy consumption could be reduced by replacing passive aeration with active aeration methods, such as using an air cathode. However, as the catholyte also acted as the DS in OsMFCs, air cathodes were not suitable. Unfortunately, no research was found on reducing the energy consumption associated with cathode aeration in OsMFCs. Furthermore, although the recovered electricity had certain economic value, the investment cost of the systems was substantial. For example, electrodes required platinum catalysts to enhance the efficiency of oxygen reduction. To enhance the eco-friendliness and sustainability of the systems, Al-mamun et al. explored an alternative approach by replacing costly catalysts with electrochemically active aerobic marine biofilms on the cathode electrode. The maximum output power and water flux of the system were 28.9 Wm^−3^ and 1.46 LMH, respectively [44].

### 3.3. Water Recovery

Compared to traditional MFCs, OsMFCs equipped with FO membranes as separators demonstrated the capability of producing high-quality water. Relevant studies have shown that more than 50% of clean water could be extracted from various wastewater by OsMFCs, which means at least 50% of water recovery could be achieved from wastewater [28].

Table 2 summarizes the studies on water recovery. The most influential factor affecting the efficiency of water recovery was the DS, including the types and concentrations of the DS. Increasing the concentration of the DS enhanced the osmotic pressure difference between the FS and DS, leading to an increase in water production for the same type of DS. In comparison with traditional FO technology, the ideal DS for OsMFCs should possess higher osmotic pressure, lower RSF, and higher conductivity to meet electricity generation requirements. Ge et al. demonstrated that the NaCl draw solution exhibited higher water flux compared to CaCl_2_ at the same concentration, despite the latter having higher osmotic pressure. This was attributed to the reverse diffusion of Ca^2+^ formed inorganic scale on the surface of FO membrane, resulting in a decrease in water flux. In addition, when PPB was used as the DS, the water flux increased significantly with the increase in PPB concentration [64]. It is worth mentioning that the recovery of clean water from the diluted DS and the reconcentration of the DS necessitated additional treatment technologies, such as RO, consequently increasing operational costs. Hence, the responsive draw solutions were employed in OsMFCs which significantly reduced the energy requirement during water recovery processes. The pH-responsive draw solution has a good buffer capacity, and their solubility could be manipulated by pH that was varied attributable to electrochemical reactions. Wang et al. employed EDTA-Na_2_ as the DS, achieving a water flux of 1.82 LMH at a concentration of 0.25M. Furthermore, the recovery rate of EDTA-Na_2_ could reach 90% by adjusting the pH [72]. Similarly, Yang et al. utilized PAA-Na as the DS, obtaining a water flux of 12.7 LMH, and a high DS recovery rate of 99.86% at a concentration of 32 wt% [71]. Furthermore, the fouling of FO membranes could significantly impact water flux [63]. Although membrane fouling was inevitable in all membrane processes [73], it particularly affects OsMFCs by causing biofouling and inorganic scaling on the FO membrane surface, resulting in reduced water flux and production rates [55]. Regular membrane cleaning was a viable option, but the cleaning process was more difficult and increased operating costs.

## 4. Operational Factors of OsMFCs

### 4.1. Configurations of OsMFCs

**Figure 5:**
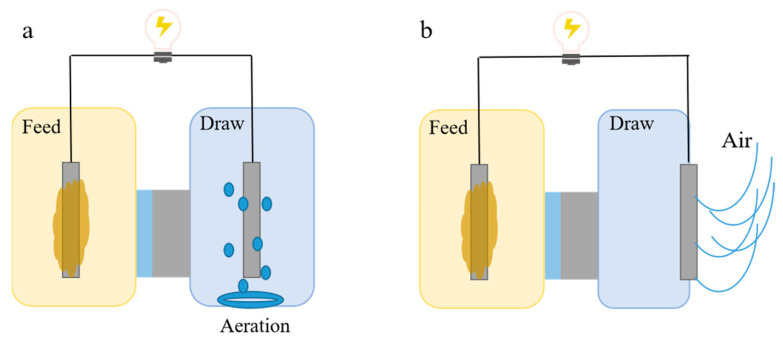
Construction of two-chamber OsMFCs (**a**) and air-cathode OsMFCs (**b**).

Due to the introduction of the FO membrane, OsMFCs necessitates extracting water from the FS to ensure a certain proton transfer and achieve water recovery, so the single-chamber configuration was not feasible. The cathode of two-chamber OsMFCs required electron acceptor and could be aerated to facilitate the reduction in oxygen [41] (Figure 4a). However, cathode aeration consumed additional energy, which could offset the electrical energy generated by the system. Another approach was the adoption of an air cathode configuration, which allowed direct contact between the cathode and air, thereby saving energy consumed by aeration (Figure 5b). Werner et al. demonstrated successful organic matter removal (90%) and enhanced power generation (43 W/m^3^) in an air cathode-type OsMFC [45]. Therefore, air cathode OsMFC was more cost feasible compared with aerated OsMFC.

### 4.2. The FO Membrane

#### 4.2.1. Types of Membrane

The parameters and properties of different FO membrane are also different. Currently, two asymmetric commercial FO membranes (Thin-Film Composite membrane (TFC) and cellulose triacetate (CTA) membranes) are widely used. Numerous studies compared the properties of TFC and CTA membranes in FO systems. Many studies on FO compared the properties of TFC and CTA membranes. Compared to CTA membranes, TFC membranes exhibited higher water flux, lower reverse salt flux, wider pH range, and better physical and chemical stability [75,76,77,78]. Membrane fouling on TFC membranes was generally more reversible and easier to remove through physical cleaning techniques [79]. However, some studies reported contrasting conclusions regarding membrane performance, which may be attributed to differences in operating conditions, such as feed and draw solution type and concentration.

Several recent studies evaluated different commercial FO membranes for their suitability in OsMFCs [31,49]. These studies indicated that the cellulose triacetate-embedded polyester screen support (CTA-ES) membrane demonstrated superior performance in terms of electricity generation and water flux, attributed to its high microbial enrichment and low oxygen permeability. However, it exhibited a higher RSF. Conversely, in a series of performance tests, polyamide (PA) membranes exhibited advantages in terms of reverse salt flux, water flux, and proton transfer capability compared to CTA-ES membranes [80], while TFC membranes exhibited lower RSF, higher water flux, and better pH stability compared to CTA membranes [49,81]. However, the commercial FO membranes were not completely proficient for OsMFCs operation. To reduce the dependence on commercial FO membranes, some studies have developed FO membranes with improved performance. Pankaj et al. prepared a high-flux layer-by-layer polyelectrolyte FO membrane with a water flux of 18.43 LMH and an energy production of 0.438 kWh/m³ [70]. Subsequently, they developed a photopolymerized active layer FO membrane, and compared its performance with that of a commercial HTI (CTA) membrane [68]. Among all the prepared membranes, the power density of the membrane with the best performance could reach 237.23 ± 0.90 mW/m^2^ and the average water flux was 0.95 ± 0.71 LMH. Further research should focus on the development and industrialization of FO membranes suitable for OsMFCs.

#### 4.2.2. Membrane Orientations

The orientation of the FO membrane was a crucial factor in the performance of OsMFCs. The orientations of the FO membrane, specifically two main orientations, namely the active layer facing the feed solution (AL-FS) and the active layer facing the draw solution (AL-DS), had a significant impact on various aspects such as water and solute flux (Table 3), nutrient and substrate concentration in the anode chamber, microbial activity, and power generation. Several studies indicated that the AL-FS orientation generally led to higher electricity generation compared to the AL-DS orientation. This might be attributed to the direct interaction between the bacteria in the anode chamber and the substrates present in the FS, thereby promoting microbial activity and electron transfer. However, employing the AL-DS orientation might have advantages in terms of mitigating fouling and biofilm formation on the FO membrane surface, ultimately improving the long-term stability and durability of OsMFCs.

Regarding the AL-DS mode, certain studies reported higher water flux and RSF [31,67,68,70] (Table 3). Conversely, another study observed a lower water flux when operating an OsMFC under the AL-DS mode [82]. The disparity could potentially be attributed to the blockage of the porous support layer by microorganisms and macromolecular organic substances present in the anolyte under AL-DS mode, thus affecting water flux. The variations in water flux and RSF between different membrane orientation modes were intricate and multifaceted, likely influenced by factors such as solute molecule size, pore size of active and proppant layers, and hydrophobicity. Yang et al. suggested that the electricity generation in OsMFCs was not significantly affected by the orientation [67], while Jiang et al. reported the open circuit voltages were not significantly different under the two modes [31].

Overall, the selection of FO membrane orientation in OsMFCs was contingent upon specific application requirements and the trade-offs among performance, stability, and maintenance. The AL-FS orientation seemed to be more advantageous for maximizing power generation and microbial activity. However, further investigation was necessary to optimize and comprehend the intricate interplay between FO membrane orientation, microbial activity, and power generation in OsMFCs.

#### 4.2.3. Membrane Modification

Membrane modification has been proven as an effective strategy to improve the performances and stability of OsMFCs. By altering the surface properties of the FO membrane, modifications could mitigate membrane fouling, biofilm formation, and other detrimental factors. Several techniques for membrane modification have been investigated in relation to their impact on OsMFCs performances, including nanoparticles addition, membrane surface coating, and membrane surface chemical treatment. Yang et al. applied an FO membrane modified with nAg deposited on a polydopamine (pDA) coated membrane surface in OsMFCs [62]. The modification simultaneously reduced membrane biofouling and increased electricity generation. Moreover, the NaCl rejection rate increased to 93.8% of the modified membrane compared with 90.4% of the pristine membrane. Similarly, Lu et al. employed nAg-modified FO membranes in OsMFCs [74]. Although both water flux and RSF were reduced compared to the pristine membrane, the specific reverse solute flux (the ratio of RSF and water flux (Js/Jw), sRSF), which reflected the membrane selectivity and process efficiency was also decreased [83]. To address the issue of slow proton transport in OsMFCs, introducing sulfonic acid groups to the FO membrane as proton-conducting sites has been explored [34]. Using an optimized FO membrane in the OsMFC system, significant improvements were achieved, including a 58.86% reduction in salinity of artificial seawater, a 61.90% increase in water extraction volume, a 6.39% increase in chemical oxygen demand removal efficiency, and a 16.56% increase in current density. It is worth noting that the choice of membrane modification technique should consider the specific application requirements and involve a balance between performance, cost, and maintenance.

### 4.3. The Draw Solution

#### 4.3.1. Types of Draw Solution

The DS played a pivotal role in creating the osmotic pressure gradient essential for water transport through the FO membrane in the OsMFCs. The choice of draw solution could affect the water flux and power density of the OsMFCs, as well as the overall efficiency and cost of the system. Draw solutions could be roughly divided into inorganic and organic. Some studies have investigated the performance of various inorganic draw solutions in OsMFCs [41]. The results indicated that the Na^+^-series DS exhibited superior performance in terms of electricity generation, water flux, and RSF reduction. On the other hand, draw solutions such as NH_4_^+^-, Ca^2+^-, and Mg^2+^-series could result in solute loss through RSF, negatively impacting microbial activity during operation [84,85,86]. Among the inorganic draw solutions, K_2_SO_4_ exhibited the lowest sRSF attributed to the larger-sized SO_4_^2-^ anions. HCO_3_^-^ was an ideal pH buffer material [87]. Wu et al. utilized NaHCO_3_ as the draw solution in OsMFCs, which effectively buffered both the anode and cathode [66]. At the same conductivity with NaCl, NaHCO_3_ obtained higher electricity generation. Phosphate buffer solution (PBS) also exhibited good buffering and electricity generation [88,89,90]. However, the reverse diffusion of phosphate could lead to eutrophication in the effluent [66,91]. NaCl, being easily accessible, was often used as an extractant in various studies, but required prior acidification to manage pH changes [36,51,61,74].

Conventional organic DSs like glucose could generate osmotic pressure, but their electrical conductivity limited their application in OsMFCs [92]. Responsive draw solutions, particularly polyelectrolyte salts, have gained significant interest (as shown in Table 4). For instance, disodium ethylenediaminetetraacetate (EDTA-Na_2_) was explored as DS and exhibited comparable electricity generation and water flux to NaCl at the same conductivity, while offering lower RSF and excellent recyclability with up to 90% of EDTA being recoverable and reusable [93]. Yang et al. investigated the performance of polyacrylic acid sodium (PAA-Na) as the DS [71]. The OsMFC achieved a current density of 159 ± 6 A/m^3^, water flux of 12.7 ± 0.2 LMH, and low RSF of 0.05 g m^−2^ h^−1^ (gMH) using 32 wt% PAA-Na (2000 Da) as the DS. The recovery efficiency reached 99.86 ± 0.04% with the aid of high catholyte pH. The choice of DS was a crucial factor in determining the performance and efficiency of OsMFCs. Various considerations such as availability, cost, toxicity, and sustainability should be taken into account when selecting the optimal DS. These factors ensured that the chosen DS was not only effective but also practical in terms of its impact on both the system and the environment.

#### 4.3.2. Concentration of the Draw Solution

The concentration of the DS was an important parameter that could significantly affect the performances of OsMFCs. Increasing the draw solution concentration results in a higher osmotic pressure difference across the membrane. However, it was crucial to recognize that there existed a threshold beyond which elevating the DS concentration might yield adverse effects on OMFC performance. It was primarily due to the potential escalation in costs and an associated increase in RSF. Qin et al. demonstrated that increasing the DS concentration from 2 g/L to 35 g/L resulted in an increase in current density [60]. The increase in electricity production was attributed to enhanced water flux, which promoted proton transfer. However, it should be noted that the electrical performance did not exhibit an unlimited increase with the concentration of the DS. He et al. proposed that the relationship between the concentration of the DS and current density followed a curve, where after the inflection point, further increases in concentration did not lead to a subsequent rise in current density [94]. Additionally, the initial DS concentration had a negligible effect on chemical oxygen demand (COD) removal efficiency. Therefore, careful optimization of the DS concentration was essential in OsMFCs to achieve the highest power output and efficiency while considering factors such as membrane fouling and electron transfer resistance.

### 4.4. The Feed Solution

In laboratory studies, the anolyte typically consists of synthetic wastewater, utilizing organic compounds such as glucose and sodium acetate as microbial carbon sources [66,70]. However, artificial carbon sources were not sustainable in practical applications. Actual domestic wastewater was utilized as a microbial carbon source in OsMFCs, resulting in a COD removal rate of 92%, a power density of 48.52 mW/m^2^, and a current density of 136.30 mA/m^2^ [63]. The salt content of the feed solution was also one of the issues that had to be considered. High salt wastewater has a negative effect on the electrical performance of anode microorganisms. However, adding ectoine to the anolyte could enhance the salt tolerance of microorganisms, resulting in a voltage output increase of 60.4 ± 3.9% [95].

The microbial composition of the feed solution also greatly influenced the performance of OsMFCs [96]. Electroactive microbes, such as *Shewanella* and *Geobacter*, played a crucial role in converting the chemical energy of wastewater into electricity. These microbial species have been extensively studied for their extracellular electron transfer mechanisms [97]. In addition, *Clostridium* and *Pseudomonas* were also commonly inoculated in BES as electrogenic microorganisms [98]. Different strains exhibit varied effects on the performances of OsMFCs. Cao et al. compared the electrical generation performance of three specific strains in bioanode electrodes and found that *S. oneidensis* outperformed *P. delhiensis* and *P. citronellolis*, producing a higher power density of 87 mW/m^2^ and a higher current density of 747 mA/m^2^ [99].

### 4.5. Other Factors

Hydraulic retention time (HRT) was also an important parameter that affects the performances of OsMFCs during operation. Prolonged HRT could lead to an accumulation of anodic liquid salt, resulting in reduced water flux and decreased electrical production performance [100,101,102]. When the HRT was extended from 3.0 h to 6.0 h, the water flux decreased from 1.64 ± 0.09 LMH to 1.52 ± 0.11 LMH, and the electricity production decreased from 218.1 ± 9.5 mV to 141.0 ± 16.2 mV. Therefore, a lower HRT was beneficial for alleviating salt accumulation in the anolyte and improving the electrical generation. However, reducing the HRT negatively impacted the degradation of COD and contaminants in the anolyte [103,104], which was attributed to the reduced interaction time between these substances and microorganisms. For example, a COD removal efficiency of 50–55% was observed at an HRT of 3 days, compared to 85–90% at an HRT of 6 days [105]. Therefore, the HRT should be adjusted based on the specific requirements of wastewater treatment. A lower HRT could be set for water and electricity production, while a higher HRT should be considered for pollutant degradation.

Recently, the effects of magnetic fields on the performances have been investigated, including the direction of the magnetic field and water flow [106]. When the magnetic field was antiparallel to the water flow, OsMFC performances were improved. The maximum water flux, RSF, power density, and current density reached 0.63 ± 0.02 LMH, 3.02 ± 0.2 gMH, 26.58 ± 12 Mw/m^2^ and 266.29 mA/m^2^, respectively. Furthermore, the coulomb efficiency was improved by 20–30%, and the startup time of OsMFCs was reduced by 1–2 days due to the magnetic field. However, it should be noted that a high magnetic flux density of ≥3 mT could have adverse effects on microorganisms, and the energy required to create the magnetic field should also be considered.

## 5. Perspectives and Challenges

### 5.1. Potential Applications

The initial research on OsMFCs primarily focused on improving electricity generation performance. Subsequent studies demonstrated the feasibility of simultaneously removing contaminants from the anolyte while generating electricity. The electrochemical osmosis systems have shown promise in the recovery of metals such as gold, silver, and mercury from wastewater through hydropower co-generation [107], but there are few studies on metal recovery from wastewater at present. Syeed et al. pointed out that bio-electrochemical systems could achieve ammonium and metal removal/recovery [27]. MFCs have shown feasibility in metal recovery from wastewater, the low concentration of many metals in wastewater incurs high costs. From an economic perspective, the systems would be more viable for the recovery of high-concentration precious metals present in wastewater.

Furthermore, due to the involvement of biological processes, OsMFCs exhibited potential for the removal of emerging pollutants, such as pharmaceutical and personal care products (PPCPs), in addition to the conventional organic matter (COD) removal from wastewater. The removal rate of MFC for sulfamethoxazole (SMZ) could reach more than 85% [108], and an optimal dosage of SMZ could enhance the electrical performance of MFC [109]. MFC also had a good removal effect on other PPCPs [110,111]. Overall, OsMFCs exhibited immense potential for application in wastewater treatment and the removal of emerging pollutants, but most of the current research focused on MFC, leaving a gap in the understanding of OsMFCs. Additionally, differences existed between the internal environments of MFC and OsMFCs, necessitating further investigation into the removal mechanisms and efficiency of OsMFCs for the emerging pollutants.

### 5.2. Membrane Fouling

Membrane contamination was a common issue in membrane separation processes, including FO membranes in OsMFCs. While the fouling trend of FO membranes was relatively low compared to other pressure-driven membrane separation processes [112], it still affects the membrane’s separation performance and lifespan, leading to a decrease in membrane flux [113]. Biological pollution, caused by microorganisms, played a more significant role in the formation of the fouling layer on the membrane [25]. Researchers has explored methods to mitigate biological fouling in OsMFCs. Lu et al. modified FO membranes by using silver nanoparticles (AgNP), which directly interfered with the function of microorganisms deposited on the surface of the membranes, effectively alleviating the biological fouling of FO membranes in OsMFCs [74,114]. However, membrane fouling was a relative concept. Zhu et al. found that mild membrane fouling could induce proton and other ion diffusion, increase transmembrane ion flux, decrease internal resistance, and improve current generation [54]. In addition to modifying the membrane by physical and chemical means to prevent membrane fouling [51,115], we should also pay attention to the timing of removing membrane fouling and retaining part of the effective membrane pollution. More efforts are needed to develop sustainable biofouling prevention and cleaning technologies to extend the service life of membranes.

### 5.3. System Expansion

OsMFCs could achieve sustainable water treatment and energy production. However, the efficiency and scale of this technology are still insufficient for practical application. Scaling up reactors in OsMFCs requires a consideration of cost issues, as energy consumption typically accounts for a significant portion of operating costs [2]. The cost of FO membranes, which constitute a significant portion of the system construction and expansion costs, is a critical consideration. Reducing the cost of FO membranes or even finding alternatives would improve the feasibility of scaling up the systems. Recently, laminar microfluidic method has been used to construct membrane-less OsMFCs with a maximum power density of 87 Mw/m^2^ and a current density of 747 mA/m^2^ [99]. Moving the OsMFCs from the laboratory to the practical application still needs to overcome the problems of low water-treatment efficiency and capacity efficiency, high cost, and ensure the stability of scaled-up systems.

### 5.4. Reverse Solute Flux

When the proton exchange membrane was replaced with an FO membrane in OsMFCs, the overall performances were greatly improved due to the higher proton transfer efficiency of the FO membrane [30,49]. However, the issue of RSF in FO was inevitable, which led several detrimental effects, including DS loss, reduced effluent quality, decreased osmotic pressure, and increased treatment costs [37,38,116]. Additionally, RSF could negatively affect the activity of functional microorganisms in bioreactors [117,118]. Therefore, it was necessary to adopt various strategies to inhibit RSF, and these strategies have been comprehensively summarized and analyzed in a previous review [119].

Some studies found that power generation could significantly reduce the RSF of cations and anions, and the decrease in the ratio of anions to RSF might be caused by the synergistic effect of electric field, FO film, and charge balance [41]. While RSF has traditionally been considered to have a negative effect on microbial activity and power generation performance, its potential positive effects have been ignored. Using NaHCO_3_ solution as the draw solution, the reverse flux of NaHCO_3_ helped buffer the pH of the anolyte, thereby increasing the power generation [66,120]. The reverse diffusion characteristics of FO were utilized to recover nitrogen and phosphorus in synthetic urine by using bivalent magnesium salt solution as the draw solution [121]. In the near future, inhibiting or utilizing RSF could further improve reactor performance and facilitate the early practical application of OsMFCs.

### 5.5. Full Life-Cycle Assessment

At present, the environmental impacts of OsMFCs have not been studied. The full life-cycle assessment and cost accounting of OsMFC are still very lacking, and there is no systematic and comprehensive study on the life cycle and environmental impact of OsMFCs. Zhang et al. established an attributional life-cycle assessment model based on a laboratory scale to evaluate the environmental impact of OsMFCs from cradle to grave [122]. The results showed that the greenhouse gas emissions of OsMFCs were higher than those of conventional wastewater treatment technologies. However, this study only focused on specific OsMFCs, and further life-cycle assessments of OsMFCs constructed with different materials should be conducted. It is important to evaluate the environmental impact of pilot-scale and larger-scale OsMFCs in actual operation and conduct more comprehensive cost calculations. It would provide valuable insights for the practical operation and management of OsMFCs. Under the background of carbon emission reduction, improving the treatment efficiency of the system, increasing resource and energy recovery, and low-carbon operation should be the future development directions.

## 6. Conclusions

The research on OsMFCs have a history of just over a decade, and there has been a notable lack of attention given to their potential applications in wastewater treatment, wastewater reclamation, and energy recovery. The combination of MFC and FO could have mutual benefits. MFC facilitated the FO process by enhancing water flux, reducing RSF, and degrading contaminants in the FS. Furthermore, the water flux based on the FO principle also enhanced electricity generation in the MFCs. OsMFCs have the potential to recover nutrients, energy, and clean water from wastewater. The operational factors that affect the performance of OsMFCs include the configurations, FO membranes (types, orientations, and modifications), and DSs (types and concentrations). In addition to the removal of conventional pollutants, OsMFCs also showed promise in treating wastewater containing emerging pollutants. However, there were challenges to overcome for the commercial application of OsMFCs, such as membrane fouling, system scale-up, and RSF. Despite these obstacles, the increasing demand for better technologies, advancements in related fields, and innovative ideas might lead to breakthroughs in OsMFCs. Therefore, the future of OsMFCs remains promising.

## Figures and Tables

**Table 1:** Summary of the OsMFCs studies for energy recovery.

System	Feed Solution	Draw Solution	Maximum Power Density	Current Density or Current	Reference
OsMFC	synthetic livestock wastewater-	NaCl, 35 g/L	-	2.6 ± 0.1 Am^−2^	[60]
OsMFC	fresh urine	NaCl, 0.25 M	0.12187 Wm^−3^	-	[61]
OsMFC	artificial wastewater	NaCl, 35 g/L	61.5 ± 0.9 mWm^−2^	-	[62]
OsMFC	actual domestic wastewater	real oilfield produced water	48.52 mWm^−2^	136.3 mAm^−2^	[63]
OsMFC	synthetic wastewater	NaCl, 1 M	-	2.1 mA	[64]
		potassium phosphate buffer	-	2.5 mA	
		CaCl_2_, 1 M	-	2.2 mA	
		Glucose, 1 M	-	0 mA	
OsMFC	landfill leachate	NaCl, 1 M	0.44 W m^−2^	-	[31]
OsMFC	synthetic fresh human urine	MgCl_2_, 0.5 M	-	27.3 Am^−3^	[65]
OsMFC	synthetic municipal wastewater	NaHCO_3_, 0.75 M	-	18.0 Am^−3^	[66]
OsMFC	synthetic wastewater	NaCl, 35 g/L	-	374.7 mA m^−2^	[67]
OsMFC	synthetic wastewater	NaCl, 35 g/L	7.08 Wm^−3^	5.4 Am^−3^	[34]
OsMFC	synthetic wastewater	NaCl, 1 M	12.57 Wm^−3^	-	[50]
OsMFC	synthetic wastewater	NaCl, 35 g/L	237.23 mWm^−2^	-	[68]
CW-OsMFC	synthetic wastewater	NaCl, 2 M	59.53 mWm^−2^	360.36 mA m^−2^	[69]
OsMFC	synthetic wastewater	NaCl, 2 M	4.5 Wm^−3^	-	[28]
OsMFC	synthetic wastewater	NaCl, 0.5 M	2.62 Wm^−3^	-	[25]
OsMFC	synthetic wastewater	NaCl, 2 M	27.38 Wm^−3^	139.52 Am^−3^	[70]
OsMFC	synthetic municipal wastewater	PAA-Na, 32 wt%	-	159.0 Am^−3^	[71]

**Table 2:** Summary of the OsMFCs studies for water recovery.

System	Membrane Type	Feed Solution	Draw Solution	Water Flux (LMH)	Reference
OsMFC	TFC	synthetic livestock wastewater	NaCl, 35 g/L	1.3	[60]
OsMFC	CTA	fresh urine	NaCl, 2 M	14.27	[61]
OsMFC	CTA	artificial wastewater	NaCl, 35 g/L	2.33	[62]
OsMFC	CTA	actual domestic wastewater	real oilfield produced water	4.17	[63]
OsMFC	CTA	synthetic wastewater	NaCl, 1 M	1.82	[64]
			PPB, 1 M	2.42	
OsMFC	CTA-ES	landfill leachate	NaCl, 1 M	0.98	[31]
	CTA-NW			0.56	
	TFC-ES			0.79	
OsBCRS	TFC	synthetic fresh human urine	MgCl_2_, 0.5 M	18.4	[65]
OsMFC	TFC	synthetic municipal wastewater	NaHCO_3_, 0.75 M	3.5	[66]
OsMFC	CTA-NW	synthetic wastewater	NaCl, 35 g/L	0.57	[67]
OsMFC	modified TFC	synthetic wastewater	NaCl, 35 g/L	12.29	[34]
OsMFC	TFC	synthetic wastewater	NaCl, 1 M	6.0	[50]
OsMFC	polyelectrolyte membrane	synthetic wastewater	NaCl, 2 M	18.43	[70]
OsMFC	photopolymerized active layer FO membrane	synthetic wastewater	NaCl, 35 g/L	0.95 ± 0.71	[68]
OsMFC	TFC	synthetic wastewater	NaCl, 2 M	6.66	[69]
OsMFC	CTA	synthetic municipal wastewater	EDTA-Na_2_, 0.25 M	1.82	[72]
OsMFC	TFC	synthetic wastewater	NaCl, 0.5 M	3.57	[25]
OsMFC	TFC	synthetic municipal wastewater	PAA-Na, 32 wt%	12.7	[71]
OsMFC	modified TFC	synthetic domestic wastewater	NaCl, 0.5 M	8.5	[74]

**Table 3:** The effect of membrane orientations on OsMFCs performance (“+” means the value is higher).

Membrane	Application	Feed Solution	Draw Solution	Mode	Mode	Water Flux	RSF	Reference
CAT-ES	OsMFC	Landfill leachate	NaCl	AL-DS		+	+	[31]
				AL-FS	+			
TFC-ES				AL-DS		+	+	
				AL-FS	+			
CTA-NW				AL-DS		+	+	
				AL-FS	+			
CTA-NW	FO-BES	Synthetic wastewater	NaCl	AL-DS		+	+	[67]
				AL-FS				
Polyelectrolyte FO Membrane	OsMFC	Synthetic wastewater	NaCl	AL-DS		+	+	[70]
				AL-FS				
		DI water		AL-DS		+	+	
				AL-FS				
FO Membrane	OsMFC	Synthetic wastewater	NaCl	AL-DS				[82]
				AL-FS		+		

**Table 4:** Summary of responsive draw solution in the OsMFCs.

Membrane	Draw Solution	Current Density (A·m^−3^)	Water Flux (LMH)	RSF (gMH)	sRSF	Recovery Efficiency (%)	Reference
					(mg·L^−1^)		
CEM-FO	2000 PAA-Na (32 wt%)	159.0 ± 6.0	12.7 ± 0.2	~0.05	~0.9	>99	[71]
	PBS (8 wt%)	167.0 ± 6.0	~3.0	9.12 ± 0.10	~3000	-	
CTA-HTI-FO	EDTA-Na_2_ (0.2 M)	22.5	1.57 ± 0.5	0.38	250	>90	[72]
	NaCl (0.185 M)	23.6	1.22	0.55	450	-	

## Data Availability

No new data were created or analyzed in this study. Data sharing is not applicable to this article.
